# Speculative bubbles and herding in cryptocurrencies

**DOI:** 10.1186/s40854-022-00383-0

**Published:** 2022-08-23

**Authors:** Ozkan Haykir, Ibrahim Yagli

**Affiliations:** 1 Finance and Banking Department, Nigde Omer Halisdemir University, Nigde, Turkey; 2 Accounting and Finance Department, Nevsehir Hacı Bektas Veli University, Nevsehir, Turkey

**Keywords:** Cryptocurrency, Bubbles, Co-explosivity, Herding, COVID-19 pandemic, G12, G17, G40

## Abstract

This study investigates speculative bubbles in the cryptocurrency market and factors affecting bubbles during the COVID-19 pandemic. Our results indicate that each cryptocurrency covered in the study presented bubbles. Moreover, we found that explosive behavior in one currency leads to explosivity in other cryptocurrencies. During the pandemic, herd behavior was evident among investors; however, this diminishes during bubbles, indicating that bubbles are not explained by herd behavior. Regarding cryptocurrency and market-specific factors, we found that Google Trends and volume are positively associated with predicting speculative bubbles in time-series and panel probit regressions. Hence, investors should exercise caution when investing in cryptocurrencies and follow both crypto currency and market-related factors to estimate bubbles. Alternative liquidity, volatility, and Google Trends measures are used for robustness analysis and yield similar results. Overall, our results suggest that bubble behavior is common in the cryptocurrency market, contradicting the efficient market hypothesis.

## Introduction

Financial technology—especially payment and money transfer systems—has become quite popular owing to its considerable contribution to the financial system by lowering trading costs and improving trading quality (Kou et al. 2021). Cryptocurrency, powered by blockchain technology, is an exceptionally innovative product that has shaped financial technology in recent years. Rising public interest in cryptocurrencies and soaring cryptocurrency prices has ignited discussion in both academic and political fields. Social and economic aspects, two essential aspects in cryptocurrencies, have received considerable attention. Discussions on the social aspect predominantly focus on using cryptocurrencies in illegal transactions such as money laundering and illicit financing. Conversely, discussions on the economic aspect revolve around issues such as efficiency, diversification benefits, and price dynamics (Corbet et al. 2019; Jalal et al. 2021).

Our study concentrates on the economic aspects of the cryptocurrency market and attempts to determine whether cryptocurrencies prevail in bubble behavior, given the rapidly increasing prices during the unstable market conditions brought by the COVID-19 pandemic (Jalal et al. 2021). We also investigated co-explosivity among cryptocurrencies to understand the transmission of bubbles from one cryptocurrency to another. Furthermore, we examine determinants of the bubbles by addressing herd behavior and cryptocurrency-specific and market-related factors.

Soaring cryptocurrency prices, without any clear justification, have triggered suspicion about whether these sharp price increases represent speculative bubbles. The possibility of bubble formation in cryptocurrency prices originates from several factors. First, a bubble is characterized as a divergence between an asset’s market value and intrinsic value. Cryptocurrencies, for instance, have no fundamental value (Cheah and Fry 2015). Second, digital currency market inefficiency (Urquhart 2016; Zhang et al. 2018; Cheah et al. 2018) may cause explosive cryptocurrencies price movements. Third, bubbles are closely related to technological innovation (Pástor and Veronesi 2009; Frehen et al. 2013). Therefore, expecting cryptocurrency market bubbles is plausible owing to cryptocurrencies being financially innovative products. Fourth, cryptocurrencies seem to be speculative investments rather than real currencies (Yermack 2015). Monetary expansion during the pandemic may cause bubbles in these speculative assets’ prices. Furthermore, common beliefs regarding cryptocurrencies soon becoming a widely payment method also trigger sharp increases in cryptocurrency prices (Chaim and Laurini 2019). Fifth, the limited supply of most cryptocurrencies (e.g., Bitcoin, Cardano, and Stellar) may induce bubble formation in cryptocurrency prices. Tirole (1985) highlights that scarcity is one of three conditions for bubble formation. Cryptocurrencies’ maximum supply limit creates a suitable environment for bubble formation. Scarcity creates an over-expectation of future profits, causing speculative bubbles. Finally, many investors do not have sufficient knowledge on cryptocurrencies. A survey conducted by Cardify (2021) confirms that many investors possess a low level of cryptocurrency knowledge. Lack of financial literacy may also cause inexperienced investors to mimic others’ transactions (Bouri et al. 2019), resulting in extreme price movements.

Based on the above possibilities, our study attempts to detect bubble behavior in the cryptocurrency market during the pandemic and determine the underlying factors behind speculative bubbles. This study contributes to the literature in three ways. *First*, while bubble formation has been addressed empirically in several asset classes (e.g., stocks, currency, gold, energy, and real estate) (Johansen and Sornette 1999; Assenmacher and Czudaj 2015; Sharma and Escobari 2018; Zhang et al. 2021; among others), bubble behavior in the cryptocurrency market is an emerging field of study. Furthermore, while recent studies deal with the speculative bubbles in cryptocurrencies, they predominantly analyze the existence of bubbles in Bitcoin, the best-known and most-traded cryptocurrency (Cheah and Fry 2015; Geuder et al. 2019; Chaim and Laurini 2019). Hence, evidence of the presence of bubbles in alternative coins (altcoins) is scarce (Kyriazis et al. 2020). Unlike other studies, our study analyzes bubble formation in altcoin prices with the highest market capitalization, along with Bitcoin.

*Second*, although several studies have addressed the existence of bubbles in cryptocurrencies (Cheah and Fry 2015; Corbet et al. 2018; Geuder et al. 2019; Chaim and Laurini 2019; Enoksen et al. 2020; among others), no study has investigated bubble behavior in the cryptocurrency market amid the COVID-19 pandemic, where investing behavior has changed due to discontinued operations and measures taken by policymakers (Mandaci and Cagli 2021). COVID-19 has dramatically affected financial assets, particularly stocks; however, cryptocurrency prices, especially those of altcoins, rose sharply during COVID-19 and peaked during the pandemic period.^1^ Several studies have reported that cryptocurrencies acted as safe havens during the pandemic (Corbet et al. 2020; Demir et al. 2020; Goodell and Goutte 2021; Mariana et al. 2021). Conversely, numerous studies have revealed that cryptocurrencies are not a safe havens (Conlon and McGee 2020; Conlon et al. 2020; Będowska-Sójka and Kliber 2021). Additionally, COVID-19 is expected to trigger explosive behavior as it reduces market efficiency (Narayan 2020). Considering ambiguous evidence regarding the safe haven property of cryptocurrency and decreasing market dynamics, we investigated bubble formation in the COVID-19 pandemic.

*Third*, and most importantly, this study attempts to explore the factors behind bubbles in cryptocurrencies. Recently, Enoksen et al. (2020) investigated cryptocurrency bubble determinants by addressing volatility, transactions, volume, popularity, and uncertainty. We extend literature on the determinants of cryptocurrency bubbles in three aspects. We address whether herding behavior is one of the drivers of bubbles (Johansen and Sornette 1999). Cryptocurrencies are new and complex financial assets, and factors affecting their prices remain unclear. Furthermore, many investors have low cryptocurrency knowledge (Cardify 2021). Additionally, the cryptocurrency market is inefficient (Urquhart 2016; Zhang et al. 2018; Cheah et al. 2018), and cryptocurrency price volatility is higher than that of traditional investment tools. Furthermore, one salient property of the cryptocurrency market is its decentralized financial system; hence, no official authority protects uninformed small investors. These cryptocurrency market properties may cause uninformed investors to mimic the transactions of other market participants. Previous studies have also provided strong evidence of herding in the cryptocurrency market (Kallinterakis and Wang 2019; Vidal-Tomas et al. 2019; da Gama Silva et al. 2019; Kaiser and Stöckl 2020; Ballis and Drakos 2020; Susana et al. 2020; Papadamou et al. 2021; among others). More importantly, cryptocurrency investors tend to herd as uncertainty increases (Bouri et al. 2019). Hence, herding behavior is more likely to prevail during the pandemic when global uncertainty is extremely high.

In addition to herding, we attempt to understand the effects of other covariates on bubble behavior. Recently, cryptocurrencies have attracted considerable attention worldwide. Jalal et al. (2021) claim that cryptocurrencies’ lower transaction costs, unique peer-to-peer transaction platforms, and fewer regulations are the reasons for its popularity among investors. Rapid cryptocurrency market growth also increases the curiosity, capturing determinants of cryptocurrency returns among academics and investors. Ciaian et al. (2016) suggests that cryptocurrencies have similar underlying characteristics as equities; therefore, factors that can predict equities can be used to understand future price movements of cryptocurrencies. Based on this similarity, studies have used two sets of variables: cryptocurrency-specific and market-related. The first set captures determinants directly associated with cryptocurrencies (e.g., volatility, trading volume, or past returns). For instance, Balcilar et al. (2017) propose that trading volume is key for predicting expected return on Bitcoin. Kristoufek (2013), Panagiotidis et al. (2018), and Aalborg et al. (2018) show that Google Trends’ search volumes are associated with future cryptocurrency returns. Rohrbach et al. (2017) and Bianchi and Dickerson (2019) suggest that a momentum strategy provides abnormal return in the cryptocurrency market. The latter comprises variables associated with the overall market, such as market returns. Finally, the lack of cryptocurrency information (Cardify 2021) and no conventional fundamental pricing models for cryptocurrencies (Shahzad et al. 2022) provide a set ground for explosivity in one cryptocurrency that can be transmitted to others (Bouri et al. 2019). Therefore, we investigate cryptocurrency co-explosivity to understand how bubbles in one cryptocurrency may lead to explosive behavior in others.

The remainder of this paper is organized as follows. “Theoretical framework and literature review” section summarizes the theoretical framework and prior literature. “Data and methodology” section explains our data and methodology. “Empirical results” section presents our empirical findings, and “Robustness analysis” section provides robustness checks. Finally, “Conclusion” section provides concluding remarks and policy recommendations.

## Theoretical framework and literature review

This section is divided into three parts. In the first part, we briefly explain speculative bubbles and provide literature on cryptocurrency market bubbles. In the second, we explain herding behavior and herding types and the related literature. In the last section, we link the two concepts of speculative bubbles and herding behavior and provide theoretical information.

### Speculative bubbles

Although the term “bubble” has different definitions (Siegel 2003), these refer to an asset’s market price exceeding its underlying fundamental value (Quinn and Turner 2021), which indicates mispricing. However, not every temporary mispricing corresponds to a bubble, as bubbles represent a rapid and continuous price increase (Brunnermeier and Oehmke 2013). Bubbles occur owing to successive price increases; a preliminary increase in asset prices creates an expectation of future price increases and attracts new market investors. A new investor who believes that she profits from asset trading causes prices to rise further. However, this increase ends with high expectations being reversed; prices then face a sharp decline, causing the bubble to burst (Kindleberger 2016). Hyman Minsky developed a more detailed distinction for bubble formation and addressed the following five stages (Kindleberger and Aliber 2011). The first stage is the *displacement* stage, wherein financial innovations such as digital cryptocurrency increase expectations of future profits. Positive expectations accelerate the investment boom, leading to the *boom* stage (the second stage), wherein asset prices increase exponentially, causing assets to become overpriced, exceeding their fundamental value. The third stage is the *euphoria* stage, wherein trading becomes an investment frenzy. In this stage, even though investors are conscious of explosive behavior or become suspicious about a bubble, they believe they can sell the asset to unsophisticated investors. Therefore, asset trading is maintained in this stage. This stage is then followed by the fourth stage, *profit-taking*, wherein experienced investors reduce their investments by taking profit. Profit-taking continues if sufficient demand from inexperienced investors remains. However, prices eventually fall sharply when demand from inexperienced investors ends, causing *panic* (the fifth stage) in the market.

There is no common condition under which a bubble exists, and explosive behavior in asset prices can emerge owing to various dynamics. Brunnermeier (2016) addressed four different models to explain bubble formation. The first is the *rational bubble* model, which assumes that investors are rational and share identical information. Rational bubbles stem from expectations regarding increased asset prices. Essentially, traders hold an overvalued asset only if they expect the explosive behavior to continue. Hence, rational bubbles occur when trading opportunities are available. The second is the *asymmetric information bubble* model, wherein investors are rational but possess divergent information. Unlike the rational bubble model, there is no common belief regarding bubble behavior in this model. In this model, the main factor is a lack of common knowledge. Therefore, the *asymmetric information bubble* suggests that traders tend to hold an overvalued asset with the expectation that they can resell it for higher prices to unsophisticated investors or those with divergent expectations. The third model, *heterogeneous belief bubbles,* is attributable to investors’ divergent prior experiences. In this model, market participants share common knowledge but make different investment decisions based on their backgrounds, suggesting psychological bias. Bubble formation is more likely when heterogeneous beliefs are combined with short-selling restrictions as asset prices increase sharply. Moreover, demand from optimistic investors is not offset by pessimists’ short sales. In the fourth model, bubbles can emerge owing to *limited arbitrage*. According to efficient market hypothesis, bubble behavior does not occur because mispricing by irrational investors is offset by arbitrage. However, fundamental, noise trader, and synchronization risks inhibit rational investors from opposing irrational investors’ transactions. Essentially, limited arbitrage fails to eliminate the transactions of irrational traders, causing bubble behavior to prevail.

Owing to soaring cryptocurrency prices, several attempts have been made to identify cryptocurrency market bubbles (e.g., Kyriazis et al. 2020). For instance, Cheah and Fry (2015) investigated bubble behavior in Bitcoin and ascertained that it has no fundamental value and that its price contains bubbles. Fry and Cheah (2016) addressed the Ripple and Bitcoin bubble by adopting econophysical models. Results show that negative bubbles have prevailed in both digital currencies since 2014. Moreover, results indicate spillovers from Ripple to Bitcoin. Corbet et al. (2018) also investigate bubble behavior in Bitcoin and Ethereum. However, they could not identify any clear evidence of such persistent bubbles in the market for both Bitcoin and Ethereum. Geuder et al. (2019) conducted another study on bubble formation in Bitcoin prices. Their results indicate a recurring bubble behavior in Bitcoin prices. Chaim and Laurini (2019) analyzed bubble formation in Bitcoin prices by adopting a strict local martingale approach. Their findings demonstrate that Bitcoin prices had bubble characteristics from early 2013 to mid-2014 but not in late 2017. White et al. (2020) also stated that Bitcoin resembles a bubble event rather than a currency or security. More recently, Enoksen et al. (2020) attempted to detect bubbles in eight major cryptocurrencies, including Bitcoin. Results indicated multiple bubbles in all the cryptocurrencies studied. Additionally, several studies address co-explosivity in the cryptocurrency market. For instance, Bouri et al. (2019) study price explosivity in major cryptocurrencies. Their results indicate multidirectional co-explosive behavior in the market. Cagli (2019) analyzes explosivity in Bitcoin price and seven altcoins and reveals that all cryptocurrencies, except for Nem, exhibit explosive behavior and that explosivity in one cryptocurrency leads to explosivity in other digital currencies.

### Herding behavior

Herding behavior is defined as investors’ tendency to follow other investors’ opinions in their decision-making processes rather than their own beliefs (Bikhchandani and Sharma 2000). Herding behavior can generate speculative bubbles or market crashes via persistent deviations from fundamental asset price values.

The theoretical discussion regarding herding behavior is divided into two models: rational and irrational models. The *rational model* suggests that all individuals in the market have the same external information and act accordingly. Conversely, the *irrational model* refers indicates that individuals imitate others’ actions without any fundamental knowledge (Devenow and Welch, 1996). In financial markets, three possible explanations for rational herding behavior are available. First, the *information-driven model* claims that social activities may cause investors to make similar judgments in response to the same set of information (Shiller et al. 1984). However, investors may change investment decisions as they believe that other investors may have more information than them. This information-driven model (information cascade) is generated when other investors’ prior acts are internalized in terms of each investor and become criteria for investment decisions, as well as when the previous actions of others predominate over their ideas (Banerjee 1992). Second, a *reputation-driven model* stems from fund managers’ concerns about their performance compared with other fund managers (Scharfstein and Stein 1990; Trueman 1994; Graham 1999). Essentially, fund managers or analysts defer their analytical skills to avoid falling behind others and thus fail to outperform the average. This strategy causes fund managers to forego their knowledge, and herding ensues when they replicate first fund managers’ or analysts’ decisions (Bikhchandani and Sharma 2000). According to Scharfstein and Stein (1990), even if managers suffer from poor performance because of herding, they have valid reasons for not being behind other investment professionals. Finally, the *compensation-driven model* suggests that the policy of compensating investment managers causes herding behavior. If compensation depends on the relative performance of the fund managers relative to similar managers, manager’s incentives become distorted, resulting in the manager holding an inefficient portfolio (Roll 1992; Brennan 1993).

Several empirical studies also report that herding behavior prevails in the cryptocurrency market. For instance, Bouri et al. (2019) examined herding behavior in the digital currency market from 2013 to 2018 using cross-sectional absolute deviation (CSAD) methodology. The static model indicates no significant herding, whereas the dynamic model indicates time-varying herding in the cryptocurrency market. Furthermore, they revealed that herding behavior is stronger in periods of higher uncertainty. Kallinterakis and Wang (2019) also used the CSAD approach to detect herd behavior in December 2013 to July 2018. Their results confirm that herding is obvious in the cryptocurrency market, especially during bull markets, low volatility, and high-volume periods. Da Gama Silva et al. (2019) also analyzed herding and contagion behaviors across 50 cryptocurrencies from early 2015 to late 2018. They employed adaptations of cross-sectional standard deviation (CSSD) and CSAD approaches and Hwang and Salmon’s (2004) methodology to detect herding. Simultaneously, they used Forbes and Rigobon’s (2002) test and its extensions for the contagion effect. Their results confirmed the existence of herding during normal periods, while adverse herding occurred during extreme periods.

On the contagion effect, our results highlight contagion between Bitcoin and altcoins. Vidal-Tomas et al. (2019) adopted the CSSD and CSAD approaches and showed that herding became more prevalent during the down market. By studying the six leading digital currencies, Ballis and Drakos (2020) revealed that herding is evident in both bull and bear markets. Nonetheless, bullish market dispersion follows market movements faster compared to bearish events. Susana et al. (2020) analyzed herding behavior among 10 cryptocurrencies during both pre-pandemic and pandemic periods. They revealed that herding is common among all cryptocurrencies in normal periods; however, this disappears under up- and down-market conditions. More recently, Papadamou et al. (2021) examined herding behavior during bull and bear markets by dividing cryptocurrencies into various clubs based on capitalization. Their results indicate that herding behavior is stronger during the down periods.

### Speculative bubbles and herding behavior

In economic theory, “homo economicus” refers to an idealized individual who behaves rationally with complete information to maximize personal benefits. Essentially, “homo economicus” is a simplified model of human behavior, wherein every person in an economy aims to maximize their economic well-being by selecting strategies based on utility-maximizing goals. However, the historical crash of 1987 prompted scholars to examine the role of human psychology in the decision-making process of buying and selling financial assets (Shiller 1990; Tversky and Kahneman 1991). According to empirical and theoretical behavioral finance studies, investor psychology may contribute to speculative bubbles and excessive volatility in financial markets, hindering informational and allocative efficiency (King and Koutmos 2021). Investors choose to imitate others’ actions when they face uncertainty, which is frequently observed in financial markets. Because every investor aims to enter the market simultaneously, herding behavior generally results in high levels of price movement in financial markets (Pompian 2017).

Although different factors trigger financial market bubbles, herding behavior is considered a vital driver of bubbles (Lux 1995; Johansen and Sornette 1999). Herding in financial markets has been a focus of current behavioral finance studies (Cipriani and Guarino 2014). Herd behavior in financial markets may be driven by either rational or irrational expectations (Hirshleifer and Teoh 2003). Rational herd behavior is information-based and occurs when investors react similarly to new information about financial instruments. Apart from rational expectations, in the financial markets, three possible rational herding behavior models are defined: information-driven, reputation-driven, and compensation-driven models. However, irrational herding occurs when investors with inadequate information and poor risk assessment blindly follow others’ actions (Lin et al. 2013). For cryptocurrencies, both rational and irrational herding behaviors may cause price bubbles.

Investors comprise parts of the community, and community members affect each other’s decisions. Social media, newspapers, and blogs are other channels influencing investors’ opinions. Given this network effect, Sornette (2003) highlights that an agent should imitate the actions of the majority because prices are determined by supply and demand. Herding behavior and bubbles occurring through social interactions have also been theoretically argued by Chang (2014).^2^ Cryptocurrencies have no fundamental value, and their prices are mostly driven by popularity rather than by supply and demand factors, as in traditional currencies (Goczek and Skliarov 2019). This situation, combined with many experts’ bullish medium-term expectations (DeMatteo, 2021), may trigger sudden price increases in the cryptocurrency market by affecting individual investors’ assessments, which suggests that an information-driven herding model may trigger bubbles.

Reputation- and compensation-driven herding models may also cause speculative bubbles. DeMarzo et al. (2008) and Pompian (2017) propose a rational general equilibrium model wherein relative wealth concerns among investors can induce financial bubbles. According to this model, an investor in a network tends to mimic the investment preferences of other investors in the network to match others’ wealth. Essentially, fear of missing out (FOMO) pushes investors to invest in risky assets, causing asset prices to rise and bubbles to occur in financial assets. Although cryptocurrencies are highly speculative, their prices have experienced remarkable increases during the pandemic compared to other asset classes. For instance, Bitcoin price increased more than 600% from August 2020 to October 2021, whereas the S&P 500 increased by only approximately 30% in the same period. Such a rally in cryptocurrency prices may surpass the possible price crash, as in 2018 (Szalay 2021), causing investment managers to follow other managers to reach their profit level. Similarly, Lux (1995) indicates that actual returns may trigger fund managers to follow others. Above-average returns elicit greater optimism among fund managers, leading them to become more committed to others’ bullish opinions. Despite fund managers’ awareness that cryptocurrencies have no intrinsic value, tremendous increases in cryptocurrency prices drew fund managers and converted previously bearish fund managers into bullish ones (Urquhart 2021). Adopting bullish expectations towards cryptocurrencies by several institutional investors may encourage other fund managers to avoid falling behind, causing further increases in asset prices. Bullishness among institutional investors may also cause individual investors, who believe that the presence of institutional investors make the cryptocurrency market more stable, to further invest in cryptocurrencies.

In addition to rational models, lack of adequate cryptocurrency information (Cardify 2021) prompts investors to blindly follow others’ opinions and exposes the cryptocurrency market to pump and dump schemes. Essentially, psychological factors can affect the behavior of uninformed or unsophisticated investors while they make investment decisions. As they are not fully informed about asset fundamental values, they tend to believe in other investors’ decisions and follow them.

## Data and methodology

In this study, we aim to highlight the mechanisms that shape cryptocurrency price dynamics and understand why cryptocurrency prices soared in such a brief period. The purpose of this study is fourfold: (1) to investigate whether the cryptocurrency market contains a bubble given the soaring cryptocurrency prices; (2) to confirm whether there is herding during the cryptocurrency bubble periods if such behavior exists in their prices; (3) to detect possible co-explosivity among cryptocurrencies; and (4) to determine the factors affecting cryptocurrency bubbles. Accordingly, we adopted an integrated analytical approach in this study. First, we identified the possible bubble behavior in the 11 leading cryptocurrencies based on two criteria: (i) market capitalization and (ii) data availability for the entire horizon of our estimation. Our dataset included Bitcoin (BTC), Ethereum (ETH), Ripple (XRP), Cardano (ADA), Dogecoin (DOGE), Chainlink (LINK), VeChain (VET), Litecoin (LTC), Stellar (XLM), Theta (THETA), and Tron (TRX). These 11 cryptocurrencies constitute approximately 80% of overall market capitalization. In the second step, we investigated herd behavior in cryptocurrencies for the entire sample and bubble periods. Third, we analyzed co-explosivity among cryptocurrencies to detect whether a bubble in one cryptocurrency leads to bubbles in others. Finally, we addressed two groups of variables to explain factors behind the cryptocurrency bubble. The first group covered cryptocurrency-specific variables, such as lagged returns, volatility, and volume, while the second group included market-related variables, such as market returns and public interest (Google Trends).

While the COVID-19 pandemic dramatically affected financial markets, cryptocurrencies—especially altcoins—benefited during this period. Apart from rapidly increasing cryptocurrency prices, this study concentrated on the pandemic period and analyzed bubble behavior in the cryptocurrency market from January 1, 2020 to March 31, 2021. Each cryptocurrency’s daily price, trading volume, and market capitalization were downloaded from coinmarketcap.com. Moreover, we used Google Trends to retrieve Google Search data for each cryptocurrency. Finally, we obtained market returns from the Crypto Currencies Index.^3^

As cryptocurrencies are considered speculative assets, factors affecting the other assets’ prices can be useful tools for predicting cryptocurrencies (Glaser et al. 2014; Yermack 2015). Factors behind the cross-sectional expected returns of securities have been examined extensively in asset pricing literature. We adopted asset-pricing variables that can predict expected stock returns in the cryptocurrency context. The first set of variables included cryptocurrency-specific variables. A prominent determinant of expected returns is an asset’s prior return. Jegadeesh and Titman (1993) demonstrated that a positive and significant relationship exists between past and expected future returns. Another variable is trading volume, which is a well-known liquidity proxy. Prior studies demonstrated a positive relationship between trading volumes and subsequent returns. A large trading volume may result in higher expected returns over the next period (Crouch 1970; Epps and Epps 1976; Harris and Gurel 1986; Amihud and Mendelson 1986; Gervais et al. 2001). Finally, we used the volatility of cryptocurrencies constructed based on daily trading price range. This was proposed by Garman and Klass (1980) and has gained popularity (Molnár 2016; Bašta and Molnár 2018; Fiszeder 2018; Fiszeder et al. 2019; Enoksen et al. 2020). We calculate daily volatility as follows:

3 $$Volatility_{i,t} = \sqrt {\frac{1}{2}\left( {h_{i,t} - l_{i,t} } \right)^{2} - \left( {2log2 - 1} \right)c_{i,t}^{2} }$$

where *c*_*i,t*_ = *log*(*close*_*i,t*_) − *log*(*open*_*i,t*_), *l*_*i,t*_ = *log*(*low*_*i,t*_) − *log*(*open*_*i,t*_) and *h*_*t*_ = *log*(*high*_*i,t*_) − *log*(*open*_*i,t*_).

The second set of variables comprised market-related variables. Our first variable is market return. Markowitz’s mean–variance efficient model states that market return is a leading indicator in explaining cross-sectional expected returns (Markowitz 1959). We use the Crypto Currencies index as a market return, which is constructed using the 30 largest cryptocurrencies in the cryptocurrency market. The index has gained popularity as a proxy for cryptocurrency market returns in finance literature (Gümüş et al. 2019). Given that investor sentiment is one of the main drivers determining cryptocurrency prices (Kristoufek 2013; Chen et al. 2019; Chen and Hafner 2019), we also analyze how public interest affects bubble behavior. Rather than supply and demand factors, popularity drives cryptocurrency prices, as in the case of traditional currencies (Goczek and Skliarov 2019). For instance, Panagiotidis et al. (2018) reveal that among 21 potential drivers, search intensity is one of the most dominant variables affecting Bitcoin returns. Philippas et al. (2019) also found that increased media attention on social networks impacts the jump intensity of Bitcoin prices. This impact is greater during periods of higher uncertainty, and Google Search is one of the best proxies for investor sentiment (Da et al. 2011; Bank et al. 2011; Vlastakis and Markellos 2012; Han et al. 2018; Huang et al. 2020). Search volume of Google Trends data can be downloaded for several timescales and ranges between 0 and 100. Daily data were obtained for a maximum of 270 days. Because the analysis covers longer periods, we split the sample period into two sets of daily data. The first period covers the 1st of January 2020 to the 5th of August 2020 and the second comprises the 6th of August 2020 and 31st of March 2021. We then merged the two datasets to obtain complete time series data for Google Trends. We used the name of each cryptocurrency during our sample period to obtain Google Trends data.

Table 1 presents summary statistics of the variables. The returns of DOGE and THETA were higher than 100 percent in the sample period. Cryptocurrencies with relatively low market capitalization had greater volatility and were illiquid. The level of Google Trends is similar among cryptocurrencies, except for DOGE. This may be because of Twitter usage instead of the Google Trends data of DOGE investors (Ante 2021).

**Table 1:** Summary statistics

	BTC		ETH		XRP		ADA		DOGE		LTC	
	Mean	Std. Dev	Mean	Std. Dev	Mean	Std. Dev	Mean	Std. Dev	Mean	Std. Dev	Mean	Std. Dev
Return	0.537	4.135	0.643	5.322	0.418	7.012	0.971	6.699	1.556	19.538	0.399	5.337
Volume	24.318	0.471	23.503	0.503	21.787	0.818	20.242	1.388	19.085	1.297	22.030	0.605
Google Trend	3.554	0.418	3.839	0.379	2.914	0.718	3.385	0.477	2.342	1.259	3.516	0.570
Volatility	0.030	0.024	0.039	0.028	0.046	0.049	0.052	0.035	0.048	0.067	0.043	0.030

	LINK		VET		XLM		THETA		TRX		Panel	
Return	0.718	6.976	0.923	7.394	0.676	7.378	1.422	7.736	0.545	5.810	0.800	8.532
Volume	20.721	0.865	19.187	0.697	19.977	0.902	17.357	1.476	20.922	0.546	20.830	2.136
Google Trend	3.335	0.711	2.760	0.607	3.833	0.306	3.872	0.202	4.132	0.220	3.407	0.795
Volatility	0.057	0.039	0.063	0.041	0.048	0.039	0.065	0.040	0.043	0.034	0.049	0.042
Market return											0.502	4.333

Table 2 presents average correlations between variables. First, we determined the correlation coefficients of the variables for each cryptocurrency. We then calculated the cross-sectional average of the correlation coefficient following a methodology similar to that of Da et al. (2011) and Enoksen et al. (2020). Correlation coefficients among Google Trends, volatility, and volume were considerably high.

**Table 2:** Correlation analysis

	Return	Volume	Google Trend	Volatility	Market return
Return	1.000				
Volume	0.129	1.000			
Google Trend	0.059	0.240	1.000		
Volatility	0.110	0.563	0.220	1.000	
Market return	0.698	0.056	0.002	−0.139	1.000

## Empirical results

To achieve the aim of this study, we begin by identifying bubble periods for each cryptocurrency. We then investigate herding behavior during bubble periods and analyze whether explosivity in one digital currency leads to explosivity in others. Finally, we estimate factors behind the cryptocurrency bubble.

### Bubble estimation

Bubbles attract economists because explosive behavior dampens capital allocation in the economy by distorting market efficiency, as in the Dutch Tulip mania, the Mississippi bubble, the Internet bubble, and, more recently, the global housing bubble. Considering the negative impact of bubbles on the real economy, several attempts to detect explosive behavior in asset prices have been made. One strand of the literature attempted to measure bubbles based on a comparison between market and intrinsic values, determined based on the underlying asset’s net present value (Siegel 2003). However, calculating the capitalized value of future cash flows is quite difficult as expected cash flows may differ among investors and will continue for many years. Another approach for detecting bubbles is based on the explosive behavior characteristics of bubbles. Unit root tests, such as autoregressive unit root tests, can be employed to measure explosive behavior (Taipalus 2012; Phillips et al. 2011, 2014). The Markov-switching unit root test can also be used to detect bubbles (Hall et al. 1999). Econophysics models, such as the log-periodic power law model, are other tools that can be used to identify explosive behavior (Filimonov and Sornette 2013; Sornette and Cauwels 2014). Generally, these models concentrate on price increases rather than directly addressing asset prices and attempting to detect bubbles based on price increase rates.

In this study, we use a unit root test to detect cryptocurrency market bubbles. We then adopted Phillips et al. (2015a, b; hereafter PSY) methodology to detect possible bubbles. The PSY procedure adopts a recursive test methodology^4^ and is frequently employed in finance literature to identify explosive behavior in various asset prices, including commodity, energy, real estate, and virtual and digital currencies (Dirk and Kristoffer, 2012; Bettendorf and Chen 2013; Cheung et al. 2015; Corbet et al. 2018; Geuder et al. 2019; Enoksen et al. 2020; Li et al. 2020; among others).

PSY postulates that bubbles exhibit slightly explosive behavior, reflecting an autoregressive nature. Therefore, they can be captured using the right-tailed ADF test, where the null hypothesis states that series have a unit root ($H_{0} :\delta_{{r_{1} ,r_{2} }} = 1$) and are tested against an alternative hypothesis wherein time series have an explosive unit root ($H_{1} :\delta_{{r_{1} ,r_{2} }} > 1$). As financial bubbles periodically emerge and conventional ADF unit root tests have limited capability of discovering recurring bubbles, PSY adopts a recursive approach containing a rolling window ADF regression sample that begins with the fraction $r_{1}$ of the total sample (*T*) and ends at fraction $r_{2}$, where $r_{2} = r_{1} + r_{w}$, $r_{w} > 0$ is the rolling window size. Regression model as follows:

4 $$\Delta y_{t} = \alpha_{{r_{1} ,r_{2} }} + \delta_{{r_{1} ,r_{2} }} . y_{t - 1} + \mathop \sum \limits_{i = 1}^{k} \psi_{{r_{1} ,r_{2} }}^{i} \Delta y_{t - i} + \varepsilon_{t}$$

Here, *α*, *δ*, and *ψ* are parameters determined by the regression, and k is the lag order. $T_{w} = \left[ {T_{w} } \right]$ is the total number of observations, where [.] is the floor function.

To consistently capture multiple bubble episodes, the PSY methodology employs a supremum ADF (SADF) test. In this estimation, window size $r_{w}$ increases from $r_{0}$ to 1, where $r_{0}$ is the smallest sample window range. Conversely, 1 is the total sample size representing the largest window size in the recursion. In the SADF test, the initial point $r_{1}$ is set to 0 and the endpoint of the subsamples equals $r_{2}$, ranging from $r_{0 }$ to 1. The SADF is robust against multiple breaks and is formulated as follows:

5 $$SADF\left( {{\text{r}}_{0} } \right) = sup_{{r_{2} \in \left[ {{\text{r}}_{0} ,1} \right] }} ADF_{ 0}^{{r_{2} }}$$

The SADF procedure is then recursively performed to construct a generalized supremum ADF (GSADF). The GSADF test allows window width to change to a predefined range by extracting more fractions of the entire sample. Therefore, this test is more flexible for determining multiple bubbles. Initial point $r_{1}$ in GSADF ranges from 0 to $r_{2} - r_{0}$ where $r_{2} \in \left[ {{\text{r}}_{0} , 1} \right]$, and the endpoint of the subsamples equals $r_{2}$ and ranges from $r_{0 }$ to 1, and GSADF is defined as follows:

6 $${\text{GSADF}}\left( {r_{0} } \right) = sup_{{\mathop {{\text{r}}_{1} \in \left[ {0,{\text{r}}_{2} - {\text{r}}_{0} } \right]}\limits^{{r_{2} \in \left[ {{\text{r}}_{0} , 1} \right]}} }} ADF_{{r_{1} }}^{{r_{2} }}$$

To date-stamp the origin and endpoints of financial bubbles, we also applied the backward supremum ADF (BSADF) test. The BSADF procedure employs either a fixed initial origin, as in SADF, or adjustable starting and endpoints. In the BSADF test, the initial point of the bubble is displayed as $T_{{r_{e} }}$ where series crosses over the critical value, and the termination of the bubble is represented by $T_{{r_{f} }}$ where series crosses the critical value downward. Estimates of the bubble period based on the GSADF are as follows:

7 $$\check{R}_{e} = \mathop {r_{2} \in \left[ {r_{0} ,1} \right]}\limits^{inf} \left\{ {r_{2} :BSADF_{{r_{2} }} \left( {r_{0} } \right) > cv_{{r_{2} }}^{{\beta_{T} }} } \right\}$$

8 $$\check{r}_{f} = \mathop {r_{2} \in \left[ {\check{r}_{e} ,1} \right]}\limits^{inf} \left\{ {r_{2} :BSADF_{{r_{2} }} \left( {r_{0} } \right) > cv_{{r_{2} }}^{{\beta_{T} }} } \right\}$$

where $cv_{{r_{2} }}^{{ _{ } }}$ is the critical value of the subsample $r_{2}$, and $\beta_{T}$ is the significance level that depends on the size of the total sample *T*. BSADF(r_0_) for $r_{2} \in \left[ {{\text{r}}_{0} , 1} \right]$ is the BSADF statistics that relate to the GSADF statistic by the following relation:

9 $$GSADF(r_{0} ) = \mathop {r_{2} \in \left[ {r_{0} ,1} \right]}\limits^{sup} \left\{ {BSADF_{{r_{2} }} \left( {r_{0} } \right)} \right\}$$

**Fig. 1:**
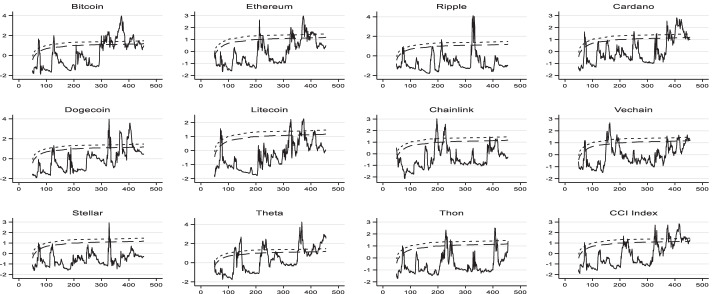
PSY bubble graphs. The small dashed line indicates the 95 percent level of critical value, while the large dashed line presents the 90 percent level of the critical value of the bootstrapped Dickey-Fuller test statistics. The straight line represents the BSADF test statistics

To identify explosive behavior in the 11 cryptocurrency prices with the highest market value, we perform a date-stamp GSADF test. Figure 1 depicts the PSY test results for each cryptocurrency. A small (large) dashed line indicates the 95 (90) percent level of the critical value of the bootstrapped Dickey-Fuller test statistics. The bubble was defined as a PSY test results (straight line) exceeding critical values. Most bubbles occurred in short windows. Table 3 also presents the number of bubble days for each cryptocurrency and its percentage in the sample. THETA, BTC, and ADA are the top three cryptocurrencies that experienced more bubble days. Conversely, XLM, XRP, and TRX are the bottom three cryptocurrencies with fewer bubble periods. When focusing on the percentage of bubble periods, cryptocurrencies tend to experience explosive behavior in 2021 compared with 2020. For instance, DOGE has more than 3 bubble days in 2021 compared with 2020. Both Fig. 1 and Table 3 emphasize that bubbles existed in all cryptocurrencies during the COVID-19 pandemic. These results are in line with previous studies presenting evidence of the presence of bubbles in cryptocurrencies (Cheah and Fry 2015; Geuder et al. 2019; Chaim and Laurini 2019; Kyriazis et al. 2020; Enoksen et al. 2020). Hence, we can proceed with our analysis to understand investors’ behavior during bubble periods by questioning herding behavior.

**Table 3:** Statistics of the bubble period

	BTC	ETH	XRP	ADA	DOGE	LTC	LINK	VET	XLM	THETA	TRX	Sum	CCI
*Panel A: number of bubble days*													
2020	48	12	15	10	9	14	21	22	4	44	13	212	10
2021	36	32	0	48	31	9	1	6	0	44	7	214	41
Sum bubble days	84	44	15	58	40	23	22	28	4	88	20	426	51
*Panel B: percentage of bubble days (%)*													
2020	15.23	3.81	4.76	3.17	2.86	4.44	6.67	6.98	1.27	13.97	4.13	6.12	3.17
2021	40	35.56	0.00	53.33	34.44	10.00	1.11	6.67	0.00	48.89	7.78	21.62	45.56
Sum bubble days	20.74	10.86	3.70	14.32	9.88	5.68	5.43	6.91	0.99	21.73	4.94	9.56	12.59

### Herding estimation

During explosive price movement periods, investment decisions tend to be affected by collective market behavior. Considering the extreme price movements in the cryptocurrency market during the COVID-19 pandemic, herding behavior may be associated with this explosive behavior. To detect herding behavior, prior studies referred to two widely used proxies. Christie and Huang (1995) proposed the first model, cross-sectional standard deviation (CSSD), and Chang et al. (2000) proposed the second model, cross-sectional absolute deviation (CSAD). Outliers easily affect the CSSD measure (Economou et al. 2011), and the CSSD model is suitable for a linear relationship between market returns and CSSD of returns (Dhall and Singh 2020). Chang et al. (2000), Mobarek et al. (2014), and Ballis and Drakos (2020) suggest that Newey and West’s (1987) standard error correction should be used to adjust estimation for autocorrelation and heteroskedasticity. Therefore, we decided to use the Newey-West standard error-corrected CSAD as our primary herd measure as follows:

10 $$CSAD_{t} = \alpha + \alpha_{1} \left| {R_{m,t} } \right| + \alpha_{2} R_{m,t}^{2} + \varepsilon_{t}$$

where *R*_*m,t*_ is the return of the CCI-30 index return, and *CSAD*_*t*_ is the return dispersion proxy, which is also determined as follows:

11 $$CSAD_{t} = \frac{1}{N}\sum\nolimits_{i = 1}^{N} {\left| {R_{i,t} - R_{m,t} } \right|}$$

Here, *R*_*i,t*_ is the return of cryptocurrency *i* at time *t*, *R*_*m,t*_ is the return on the CCI-30 index, and *N* is the number of cryptocurrencies in the portfolio. To detect herding in the cryptocurrency market during the bubble, we modified our basic model as follows:

12 $$CSAD_{t} = \alpha + \alpha_{1} \left| {R_{m,t} } \right| + \alpha_{2} R_{m,t}^{2} + \alpha_{3} R_{m,t}^{2} * Bubble_{t} + \varepsilon_{t}$$

where *Bubble* is a dummy that gets one if the bubble period and 0 otherwise. The coefficient of interest is $\alpha_{3}$ which should be significant and negative if herding behavior exists during the bubble period.

Table 4 presents the results of herding behavior analysis. Each column reports the estimation results of Eq. (12). For each cryptocurrency, we use a dummy variable to identify bubble periods. The dummy variable corresponds to bubbles in the underlying cryptocurrency, and the results determine whether herding behavior exists in the overall market. For instance, in the first column, we generated a dummy variable when there is a Bitcoin bubble and analyzed whether herding behavior exists in the entire sample. The same applies to other cryptocurrencies.

**Table 4:** Herding behavior—CSAD

Cross-sectional absolute deviation												
	BTC	ETH	XRP	ADA	DOGE	LTC	LINK	VET	XLM	THETA	TRX	CCI
Constant	0.017^a^ (0.000)	0.021^a^ (0.000)	0.017^a^ (0.000)	0.018^a^ (0.000)	0.021^a^ (0.000)	0.016^a^ (0.000)	0.019^a^ (0.000)	0.016^a^ (0.000)	0.017^a^ (0.000)	0.020^a^ (0.000)	0.016^a^ (0.000)	0.016^a^ (0.000)
$\left| {R_{m,t} } \right|$	0.525^a^ (0.000)	0.241^a^ (0.000)	0.486^a^ (0.000)	0.472^a^ (0.000)	0.199^a^ (0.000)	0.556^a^ (0.000)	0.330^a^ (0.000)	0.555^a^ (0.000)	0.516^a^ (0.000)	0.245^a^ (0.001)	0.535^a^ (0.000)	0.537^a^ (0.000)
$R_{m,t}^{2}$	−3.469^a^ (0.000)	−0.535^b^ (0.021)	−2.371^a^ (0.004)	−2.561^a^ (0.000)	−0.417^b^ (0.012)	−3.292^a^ (0.002)	−0.736^a^ (0.001)	−3.136^a^ (0.000)	−2.585^a^ (0.006)	−0.531^b^ (0.018)	−2.938^a^ (0.000)	−3.305^a^ (0.000)
$R_{m,t}^{2} * Bubble_{t}$	2.334^a^ (0.000)	3.153^b^ (0.010)	1.305^b^ (0.027)	1.520^a^ (0.001)	6.963^a^ (0.000)	2.075^b^ (0.007)	9.496 (0.251)	1.923^a^ (0.001)	1.453^b^ (0.026)	4.490^a^ (0.000)	1.762^a^ (0.001)	2.140^a^ (0.000)

*R*_*m,t*_ is the return of the CCI-30 Index, and *Bubble* is a dummy that is equal to 1 during the bubble period and 0 otherwise. Standard errors were corrected with appropriate lags based on the Newey and West (1987) method

P-values are provided in parentheses

a, b, and c represent the significance at the 1, 5, and 10 percent level, respectively

The negative and significant coefficients of $R_{m,t}^{2}$ indicates that herding behavior exists in each cryptocurrency. This result is consistent with the literature on herding behavior in the cryptocurrency market (Bouri et al. 2019; Kallinterakis and Wang 2019; Kaiser and Stöckl 2020). However, we found striking results during the bubble period. Coefficients of $R_{m,t}^{2} {*}Bubble_{t} { }$ are positive and statistically significant, suggesting that herding behavior diminishes in the overall market when a particular cryptocurrency has a bubble. This may contradict the common expectation that, during the bubble period, investors follow the crowd and invest accordingly instead of their strategies. However, this result does not indicate the opposite; rather, it indicates adverse herding owing to higher risk aversion during extreme periods (da Gama Silva et al. 2019). This result is also consistent with previous studies suggesting that herding behavior is obvious during normal periods, whereas it disappears during up and down periods (Susana et al. 2020). Supporting these findings are Vidal-Tomas et al. (2019) and Papadamou et al. (2021), who state that herding in the cryptocurrency market is stronger during down periods as most cryptocurrencies have experienced extreme price increases during the pandemic.

As a robustness analysis, we follow the literature and estimate herding behavior using CSSD and CSAD with generalized autoregressive conditional heteroscedasticity (GARCH) models as follows:

13 $$CSSD_{t} = \alpha + \alpha_{1} \left| {R_{m,t} } \right| + \alpha_{2} R_{m,t}^{2} + \varepsilon_{t}$$

where *R*_*m,t*_ is the return of the CCI-30 index return, and *CSSD*_*t*_ is the return dispersion proxy, which is also determined as follows:

14 $$CSSD_{t} = \sqrt {\frac{{\sum\nolimits_{i = 1}^{N} {\left( {R_{i,t} - R_{m,t} } \right)^{2} } }}{N - 1}}$$

where *R*_*i,t*_ is the return on cryptocurrency *i* at time *t*, *R*_*m,t*_ is the return on the CCI-30 index, and *N* is the number of cryptocurrencies in the portfolio. To detect herding in the cryptocurrency market during the bubble, we use the following basic model:

15 $$CSSD_{t} = \alpha + \alpha_{1} \left| {R_{m,t} } \right| + \alpha_{2} R_{m,t}^{2} + \alpha_{3} R_{m,t}^{2} * Bubble_{t} + \varepsilon_{t}$$

Here, *Bubble* is a dummy that is equal to 1 during the bubble period and 0 if otherwise. $\alpha_{3}$, the coefficient of interest, should be significant and negative if herding behavior exists during the bubble period.

Table 5 presents the results of the CSSD measure of herding behavior using Eq. (15). Although issues are using CSSD, as mentioned earlier, coefficients of interest in Table 5 are similar to those in Table 4. Herding behavior declines during bubble periods in most cryptocurrencies.

**Table 5:** Herding behavior—CSSD

Cross-sectional standard deviation												
	BTC	ETH	XRP	ADA	DOGE	LTC	LINK	VET	XLM	THETA	TRX	CCI
Constant	0.027^a^ (0.000)	0.032^a^ (0.000)	0.027^a^ (0.000)	0.028^a^ (0.000)	0.034^a^ (0.000)	0.026^a^ (0.000)	0.031^a^ (0.000)	0.025^a^ (0.000)	0.026^a^ (0.000)	0.032^a^ (0.000)	0.026^a^ (0.000)	0.026^a^ (0.000)
$\left| {R_{m,t} } \right|$	0.807^a^ (0.000)	0.385^b^ (0.024)	0.793^a^ (0.002)	0.723^a^ (0.001)	0.251^a^ (0.005)	0.935^a^ (0.001)	0.506^a^ (0.001)	0.928^a^ (0.001)	0.861^a^ (0.006)	0.378^b^ (0.021)	0.862^a^ (0.001)	0.835^a^ (0.000)
$R_{m,t}^{2}$	−5.483^a^ (0.000)	−1.004^c^ (0.000)	−4.276^b^ (0.027)	−4.053^a^ (0.006)	−0.648^c^ (0.053)	−6.119^a^ (0.006)	−1.270^b^ (0.010)	−5.774^a^ (0.006)	−4.802^b^ (0.044)	−0.966^c^ (0.069)	−5.081^a^ (0.006)	−5.333^a^ (0.001)
$R_{m,t}^{2} * Bubble_{t}$	3.591^a^ (0.000)	4.357^c^ (0.080)	2.400^c^ (0.066)	2.312^b^ (0.018)	13.534^a^ (0.002)	3.937^b^ (0.013)	17.98 (0.258)	3.610^b^ (0.011)	2.775^c^ (0.086)	6.823^b^ (0.012)	3.047^b^ (0.013)	3.378^a^ (0.001)

*R*_*m,t*_ is the return of the CCI-30 Index, and *Bubble* is a dummy that is equal to 1 during the bubble period and 0 otherwise. Standard errors were corrected with appropriate lags based on the Newey and West (1987) method

P-values are provided in parentheses

a, b, and c represent the significance at the 1, 5, and 10 percent levels, respectively

We also offer another widely used estimation for herding behavior to eliminate sample heteroscedasticity. Goyal and Santa-Clara (2003) demonstrate that the herding coefficient of the CSAD regression captures the link between idiosyncratic volatility and market returns. Therefore, controlling for the effect of volatility on cryptocurrency returns using the GARCH model is vital. Specifically, we added a conditional variance variable to the CSAD mean equation model and estimated the following GARCH (1,1) mean model:

16 $$CSAD_{t} = \alpha + \alpha_{1} \left| {R_{m,t} } \right| + \alpha_{2} R_{m,t}^{2} + \alpha_{3} R_{m,t}^{2} * Bubble_{t} + \theta_{1} \sigma_{t}^{2} + \varepsilon_{t}$$

17 $$\sigma_{t}^{2} = \omega_{0} + \omega_{1} \varepsilon_{t - 1}^{2} + \omega_{2} \sigma_{t - 1}^{2}$$

where *Bubble* is a dummy equal to 1 during the bubble period and 0 otherwise. $\sigma_{t}^{2}$ is the conditional variance of the residual *CSAD*_*t*_.

Table 6 presents the estimation results for herding behavior using the GARCH (1,1) model in the mean equation. Our results are consistent with the CSAD and CSSD using the Newey-West standard error models. Therefore, we conclude that the herding behavior of the overall cryptocurrency market significantly diminishes during the cryptocurrency market bubbles. The results of the herding analysis led us to explore the contemporaneous relationship between cryptocurrencies during bubble periods.

**Table 6:** Herding behavior—GARCH

Cross-sectional absolute deviation with GARCH												
	BTC	ETH	XRP	ADA	DOGE	LTC	LINK	VET	XLM	THETA	TRX	ALL
Constant	0.013^a^ (0.000)	0.015^a^ (0.000)	0.012^a^ (0.000)	0.013^a^ (0.000)	0.016^a^ (0.000)	0.012^a^ (0.000)	0.016^a^ (0.000)	0.012^a^ (0.000)	0.012^a^ (0.000)	0.015^a^ (0.000)	0.012^a^ (0.000)	0.012^a^ (0.000)
$\left| {R_{m,t} } \right|$	0.561^a^ (0.000)	0.427^a^ (0.000)	0.633^a^ (0.000)	0.565^a^ (0.000)	0.129^a^ (0.005)	0.643^a^ (0.000)	0.418^a^ (0.000)	0.650^a^ (0.000)	0.661^a^ (0.000)	0.438^a^ (0.000)	0.648^a^ (0.000)	0.637^a^ (0.000)
$R_{m,t}^{2}$	−2.505^a^ (0.000)	−0.983^a^ (0.000)	−3.202^a^ (0.000)	−2.442^a^ (0.000)	−0.148 (0.625)	−3.332^a^ (0.000)	−0.823^a^ (0.000)	−3.354^a^ (0.000)	−3.472^a^ (0.000)	−1.014^a^ (0.000)	−3.375^a^ (0.000)	−3.333^a^ (0.000)
$R_{m,t}^{2} * Bubble_{t}$	1.355^a^ (0.000)	0.572^c^ (0.063)	1.784^a^ (0.000)	1.104^a^ (0.000)	8.256^a^ (0.000)	1.799^a^ (0.000)	−1.664 (0.658)	1.894^a^ (0.003)	1.991 (0.760)	0.475 (0.564)	1.924^b^ (0.039)	1.904^a^ (0.000)
ARCH (1,1)	2.244^a^ (0.000)	2.309^a^ (0.000)	2.241^a^ (0.000)	2.206^a^ (0.000)	0.113^a^ (0.000)	2.205^a^ (0.000)	2.250^a^ (0.000)	2.156^a^ (0.000)	2.185^a^ (0.000)	2.197^a^ (0.000)	2.212^a^ (0.000)	2.240^a^ (0.000)
GARCH (1,1)	0.015 (0.505)	0.016 (0.465)	0.047^c^ (0.076)	0.025 (0.340)	0.941^a^ (0.000)	0.017 (0.513)	0.013 (0.511)	0.019 (0.486)	0.024 (0.406)	0.015 (0.494)	0.023 (0.437)	0.014 (0.573)
Wald	366.29^a^ (0.000)	448.39^a^ (0.000)	323.4^a^ (0.000)	343.9^a^ (0.000)	604.6^a^ (0.000)	323.9^a^ (0.000)	485.7^a^ (0.000)	299.6^a^ (0.000)	298.5^a^ (0.000)	435.5^a^ (0.000)	309.8^a^ (0.000)	329.6^a^ (0.000)

*R*_*m,t*_ is the return of the CCI-30 Index, and *Bubble* is a dummy that is equal to 1 during the bubble period and 0 otherwise

P-values are provided in parentheses

a, b, and c represent the significance at the 1, 5, and 10 percent levels, respectively

As the empirical analysis reveals that herding behavior diminishes during the bubble period, determining whether a reverse relationship exists between herding and cryptocurrency market bubbles is important. Following Bouri et al. (2019), we estimated herding behavior using a 30-day rolling window and defined herding at a 10% significance level. Hence, we create a dummy variable equal to 1 if the rolling t-statistic on $\alpha_{2} \le - 1.645$ and 0 otherwise. Once we have a herding behavior proxy, we employ a logistic regression and analyze the impact of speculative bubbles on herding for capturing the reverse effect. We adopt a time-series logistic regression, where the dependent variable is a binary variable that is equal to 1 if there is herding behavior and 0 otherwise. The dependent variable is the 1-day lag of the bubble in each cryptocurrency. Table 7 presents the results of the reverse relationship between herding and speculative bubbles. The findings show that only the speculative bubble in DOGE, VET, and THETA impacts herding in the cryptocurrency market, whereas the bubble in major cryptocurrencies does not impact herding behavior.

**Table 7:** Reverse relationship between Herding and Bubble

	Dependent variable: herding						
BTC_t−1_	−0.008 (0.831)						
ETH_t−1_		−0.014 (0.795)					
ADA_t−1_			0.013 (0.766)				
DOGE_t−1_				0.105^b^ (0.016)			
VET_t−1_					0.122^b^ (0.011)		
THETA_t−1_						−0.087^c^ (0.079)	
TRX_t-1_							0.064 (0.313)

We estimate the 30-days rolling window of Eq. (10) and defined that herding exists at a 10% significance level. The dependent variable is a dummy variable that is equal to 1 if the rolling t-statistic on $\alpha_{2} \le - 1.645$ and 0 otherwise. The independent variables are dummy variables that are equal to 1 during the bubble period and 0 otherwise for each cryptocurrency

P-values are in parentheses

a, b, and c represent significance at the 1, 5, and 10 percent levels, respectively

### Co-explosivity analysis

As herd behavior in the cryptocurrency market diminishes during bubble periods, the reason behind the bubbles in cryptocurrencies needs further investigation. Given that most cryptocurrencies facilitate similar technology and mining processes, the absence of fundamental techniques for calculating cryptocurrency value and low financial knowledge among cryptocurrency traders suggests that a bubble in one cryptocurrency can be transmitted to another (Bouri et al. 2019). Therefore, we explore co-explosivity in the cryptocurrency market to understand how bubbles in one cryptocurrency lead to explosive behavior in others.

We follow the procedure by Bouri et al. (2019) to investigate co-explosive price movements across cryptocurrencies by employing logistic regression after identifying bubble days in the prior section:

18 $$\log \left( {\frac{{P\left( {Y = 1|X} \right)}}{{1 - P\left( {Y = 1|X} \right)}}} \right) = \beta_{0} + \beta_{i} X_{i,t - 1} + \varepsilon_{t}$$

where the dependent variable is a dummy variable *Y* equal to 1 if the day is a bubble day and 0 otherwise. *X*_*i,t-1*_ is a set of ten dummy variables that takes 1 if each remaining cryptocurrency has a bubble on the previous day.

Table 8 presents the results of the co-explosivity analysis.^5^ Bubbles in XRP, LINK, LTC, and VET are least dependent on the existence of a bubble in other cryptocurrencies on the previous day. Presence of a bubble in ETH, LTC, and THETA increases the probability of a bubble in BTC, whereas the VET bubble has a negative impact. BTC, XRP, ADA, and DOGE increased the existence of a bubble in the ETH, whereas XLM and THETA had a negative effect. LTC is the only cryptocurrency that impacts the presence of bubbles in the XRP. Bubbles in DOGE are most affected by bubbles in other cryptocurrencies. ETH, XRP, LTC, THETA, and TRX increased the probability of bubble occurrence in DOGE, while XLM and VET had a negative impact. The probability of the presence of a bubble in the LTC increases when a bubble in the BTC, XRP, DOGE, and VET exists. LINK is only affected by TRX. The probability of a bubble in the VET increases with a bubble in the LTC. Regarding THETA, the probability of occurrence of the bubble increases when there is a bubble in BTC, DOGE, LINK, and TRX and decreases with ETH. Finally, the probability of a bubble in TRX increases with the presence of a bubble in ADA, DOGE, and LINK.

**Table 8:** Co-explosivity analysis

	BTC	ETH	XRP	ADA	DOGE	LTC	LINK	VET	THETA	TRX
BTC_t−1_		0.109^a^ (0.000)	−0.016 (0.550)	0.058 (0.153)	0.008 (0.792)	0.126^a^ (0.000)		−0.023 (0.455)	0.208^a^ (0.000)	0.027 (0.279)
ETH_t−1_	0.251^a^ (0.000)		0.043 (0.407)	0.171^a^ (0.000)	0.134^a^ (0.000)	−0.014 (0.564)		−0.036 (0.403)	-0.229^a^ (0.006)	0.002 (0.955)
XRP_t−1_	−0.074 (0.431)	0.153^b^ (0.035)		−0.105 (0.377)	0.132^b^ (0.034)	0.060^c^ (0.068)				
ADA_t−1_	0.037 (0.380)	0.069^a^ (0.004)	−0.063 (0.198)		0.032 (0.236)	−0.033 (0.270)		0.030 (0.428)	0.094 (0.166)	0.078^a^ (0.009)
DOGE_t−1_	0.064 (0.254)	0.163^a^ (0.000)	0.043 (0.340)	0.078 (0.119)		0.082^a^ (0.000)		−0.028 (0.500)	0.161^b^ (0.013)	0.050^b^ (0.034)
LTC_t−1_	0.395^a^ (0.000)	0.044 (0.179)	0.126^a^ (0.002)	−0.012 (0.889)	0.114^a^ (0.002)			0.117^c^ (0.050)	0.034 (0.747)	−0.025 (0.629)
LINK_t−1_								0.025 (0.637)	0.187^a^ (0.006)	0.125^a^ (0.000)
VET_t−1_	−0.099^c^ (0.056)	−0.031 (0.365)	0.008 (0.736)	0.007 (0.847)	−0.161^a^ (0.005)	0.067^b^ (0.047)	0.025 (0.645)		0.006 (0.936)	0.010 (0.765)
XLM_t−1_	−0.213 (0.348)	−0.279^a^ (0.006)	−0.028 (0.457)	−0.027 (0.861)	−0.193^b^ (0.038)	−0.031 (0.618)				
THETA_t−1_	0.184^a^ (0.000)	−0.078^b^ (0.045)		0.044 (0.279)	0.063^a^ (0.003)	−0.004 (0.814)	0.048 (0.178)	−0.003 (0.909)		0.018 (0.517)
TRX_t−1_	0.073 (0.245)	−0.053 (0.237)	0.035 (0.443)	0.196^a^ (0.001)	0.074^a^ (0.005)	−0.025 (0.500)	0.103^b^ (0.049)	−0.023 (0.702)	0.201^b^ (0.031)	
R-squared	0.276	0.514	0.375	0.204	0.391	0.395	0.047	0.026	0.107	0.245

P-values are in parentheses

a, b, and c represent the significance at the 1, 5, and 10 percent levels, respectively

Overall, co-explosivity estimation results are consistent with the finding that the presence of bubble in one cryptocurrency significantly increases with the existence of a bubble in others. This suggests that one of the factors behind the cryptocurrency market bubble is co-explosivity in the cryptocurrency market (Ang et al. 2005; Bouri et al. 2019; Cagli 2019). Based on the existing results, an investor can follow co-explosive price movements by switching from one cryptocurrency to another to gain profit.

### Bubble predictors

As cryptocurrency bubbles are largely characterized by cryptocurrency co-movements rather than herding, analyzing cryptocurrency-specific factors that can predict the occurrence of bubbles in each cryptocurrency is vital. Thus, we apply the probit model to identify factors behind cryptocurrency bubbles. We employ panel model estimation with all cryptocurrencies and estimations of time-series models for each cryptocurrency separately.

19 $$Bubble_{i,t} = \left\{ {\begin{array}{*{20}c} {1, \;i{\text{f}}\;PSY_{i,t} \left( {r_{0} } \right) > c\vartheta_{i,t} \left( {\beta_{T} } \right)} \\ {0, \;{\text{if}}\;PSY_{i,t} \left( {r_{0} } \right) < c\vartheta_{i,t} \left( {\beta_{T} } \right)} \\ \end{array} } \right.$$

We formulate time series and panel probit models as follows:

20 $$p\left( {Bubble_{t} = 1} \right) = \theta \left( {\beta x_{t - 1} } \right)$$

21 $$p\left( {Bubble_{i,t} = 1} \right) = \theta \left( {\beta x_{i,t - 1} + \vartheta_{i} } \right)$$

where $\theta \left( . \right)$ indicates a normal cumulative distribution function. $x_{t - 1}$ is a 1-day lagged variable consisting of factors that can be used to predict a bubble. $\vartheta_{i}$ is the random effect in the panel estimation. We follow Enoksen et al. (2020) and use random effects and robust standard errors clustered by cryptocurrency to eliminate autocorrelation and heteroscedasticity issues in panel estimation. In the time-series estimation, we use Newey and West’s (1987) robust standard errors. Variables are standardized by subtracting the sample mean and scaling it by standard deviation to obtain the coefficient so we can properly interpret variables’ economic impact. As mentioned, two sets of variables are available: the first set includes cryptocurrency-specific factors (i.e., lagged return, volume, and volatility) and the second comprises market-related factors (i.e., market return and Google Trends).

The bubble is given as a function of lagged return, volume, Google Trends, market return, and volatility:

22 $$\begin{aligned} Bubble_{i,t} & = \beta_{0} + \beta_{1} Lagged\;{\text{Re}} turn_{i,t - 1} + \beta_{2} Volume_{i,t - 1} + \beta_{3} Google\;Trend_{i,t - 1} \\ & \quad + \beta_{4} Market\; {\text{Re}} turn_{i,t - 1} + \beta_{5} Volatility_{i,t - 1} + \varepsilon_{i,t} \\ \end{aligned}$$

The last column of Table 9 reports the panel probit estimation, and the other columns present the results of the time-series probit estimations. Because the variables are standardized, higher coefficients represent stronger economic effect on the bubble. Positive coefficients suggest a higher probability of predicting bubbles. Conversely, a negative coefficient indicates a lower probability of predicting bubbles. Consistent with Enoksen et al. (2020), we find that both crypto-specific and market-related factors can predict cryptocurrency market bubbles. Turning to individual factors, volume, Google Trends, and volatility were positively associated with bubbles in the panel probit estimation. Conversely, the 1-day lagged return of cryptocurrency and market returns cannot predict bubbles in panel regressions. Time-series estimations indicate that Google Trends can predict bubbles as it is positive and statistically significant in 7 out of 11 cryptocurrencies. The lack of fundamental information regarding cryptocurrencies leads investors to follow public interest using Google Trends, as stated in Choi and Varian (2009), Choi and Varian (2012), Bijl et al. (2016), and Molnár and Bašta (2017). Only DOGE has a negative Google Trends coefficient. This may be attributable to DOGE being mostly dominated by Twitter owing to the activities of well-known individuals (Ante 2021). Volume is also positively associated with bubbles, and this is significant for five cryptocurrencies, which is consistent with Enoksen et al. (2020). Lagged return and market return do not have considerable effect in the time series, and volatility has ambiguous results.

**Table 9:** Probit regression

Dependent variable: bubble												
	BTC	ETH	XRP	ADA	DOGE	LTC	LINK	VET	XLM	THETA	TRX	Panel
Lagged return_t−1_	0.011 (0.873)	0.025 (0.449)	0.014^c^ (0.052)	−0.022 (0.219)	−0.006 (0.371)	0.001 (0.988)	0.056^a^ (0.002)	0.008 (0.571)	0.009^c^ (0.061)	0.047^b^ (0.022)	0.012 (0.523)	0.014 (0.113)
Volume_t−1_	0.062 (0.551)	0.404^a^ (0.000)	0.110^a^ (0.000)	0.176^a^ (0.000)	0.041^b^ (0.030)	0.035 (0.541)	0.025 (0.253)	0.015 (0.710)	−0.002 (0.736)	0.246^a^ (0.000)	0.016 (0.695)	0.142^a^ (0.000)
Google Trend_t−1_	0.226^a^ (0.000)	0.051^a^ (0.002)	0.019^c^ (0.064)	0.007 (0.586)	−0.053^b^ (0.013)	0.039^a^ (0.009)	0.023^b^ (0.012)	0.081^a^ (0.000)	0.008 (0.112)	0.054 (0.119)	0.042^a^ (0.005)	0.042^a^ (0.008)
Market return_t−1_	0.019 (0.588)	0.035 (0.112)	−0.014^b^ (0.029)	0.013 (0.464)	0.043^a^ (0.000)	0.010 (0.593)	−0.041^a^ (0.008)	0.004 (0.803)	−0.007 (0.137)	0.009 (0.615)	0.007 (0.613)	0.008 (0.386)
Volatility_t−1_	−0.045 (0.229)	−0.022 (0.209)	−0.014^c^ (0.069)	0.059^a^ (0.000)	0.051^a^ (0.000)	0.031^b^ (0.038)	−0.007 (0.541)	0.014 (0.239)	−0.003 (0.640)	0.027 (0.164)	0.034^a^ (0.003)	0.022^b^ (0.049)
Observation	406	406	406	406	406	406	406	406	406	406	406	4466
R-squared	0.293	0.404	0.270	0.404	0.452	0.266	0.138	0.246	0.234	0.308	0.247	0.228

The dependent variable is *Bubble*, which is equal to 1 during the bubble period and 0 if otherwise. The lagged return comprises a 1-day lag of the cryptocurrency return. Volume is the 1-day lag in the trading volume. Google Trends is a 1-day lag of Google Trends search volume. Market return is a 1-day lag CCI index return, and volatility is the 1-day lag of volatility. All variables were standardized. We used random effects and robust standard errors clustered by cryptocurrency in the panel estimation. In the time-series estimation, we used Newey–West’s (1987) robust standard errors

R-squared refers to the McFadden pseudo R-squared value

a, b, and c represent the significance at the 1, 5, and 10 percent levels, respectively

## Robustness analysis

Considering the complexity of human behavior and changing social settings, obtaining reasonable outcomes is difficult (Li et al. 2021). For this reason, we control whether our results are sensitive to the selection of variables, specifically the Google Trends, liquidity, and volatility proxies. We follow prior literature, use alternative liquidity and volatility measures, and reconstruct Google Trends data.

In the previous section, we combined two consecutive time series of daily Google Trends data. However, combining two consecutive datasets may not be a good proxy. To improve the reliability of the analysis, we follow prior literature and reconstruct Google Trends data, which can be downloaded for a maximum of 270 days. To create a complete time series of daily data for a longer period, we adopted the overlapping period strategy suggested by Bleher and Dimpfl (2018), which is a common methodology in finance literature (Enoksen et al. 2020; Yao et al. 2021). We downloaded two sets of 270 days of daily data and used 115 days as an overlapping period to rescale the Google Trends data to obtain one complete time-series data for each cryptocurrency.^6^

To better capture liquidity, we also use Amihud’s (2002) illiquidity measure as a liquidity proxy instead of the volume of each cryptocurrency. Amihud is one of the most common proxies for capturing liquidity. Brauneis et al. (2021) compared liquidity measures, including intraday proxies in the cryptocurrency market and suggested that the Amihud measure is the best proxy for capturing price impact compared to other low-frequency price impact proxies. The Amihud measure captures price changes per dollar of the volume unit of trade. Amihud’s (2002) measure is formulated as follows:

23 $$Amihud_{i,m} = \frac{1}{{D_{im} }}\sum\nolimits_{d = 1}^{{D_{im} }} {\frac{{\left| {\left. {{\text{Re}} turn_{id} } \right|} \right.}}{{Dvol_{id} * 10^{6} }}}$$

where *D*_*im*_ denotes 30 d. *Return*_*id*_ is the daily return, and *Dvol*_*id*_ is the dollar value of the trading volume on day *d* for cryptocurrency *i*. Amihud is considered as an illiquidity measure; thus, a low number indicates high liquidity. To construct daily measures, we use 30-days rolling windows.

Finally, to eliminate concerns about the opening and closing price of cryptocurrencies owing to the market being open for 24 h, we use one of the most common volatility measures, namely, realized (historical) volatility. Figlewski (1994) shows that the standard deviation of historical prices can be a good proxy for capturing volatility. Realized volatility was calculated based on a 30-day rolling window.

Table 10 presents panel probit estimation results using random effects and robust standard errors, clustered by cryptocurrency, as in Eq. (22). The variables were standardized. The first column presents the main results (Table 9). In the second column, we only change the Google Trends measure. In the third column, we use the Amihud measure as a proxy for liquidity instead of volume as well as the original Google Trends measure. In the fourth column, we use the overlapping estimation of the Google Trends proxy with the Amihud measure. In the fifth column, we only change the volatility measure. Following the two columns, we change the Amihud and Google Trends proxies. This method allowed us to compare new measures with our main results. Overall, the results reveal supporting evidence for our main analysis; therefore, results are not sensitive to variable selection.

**Table 10:** Robustness analysis

	Dependent variable: bubble						
	(1)	(2)	(3)	(4)	(5)	(6)	(7)
Lagged return_t-1_	0.014 (0.113)	0.018^c^ (0.050)	0.015 (0.149)	0.018^c^ (0.064)	0.021^a^ (0.004)	0.033^a^ (0.000)	0.030^a^ (0.001)
Volume_t−1_	0.142^a^ (0.000)	0.097^b^ (0.024)			0.154^a^ (0.000)		
Amihud_t−1_			−0.013^a^ (0.000)	−0.009^a^ (0.000)		−0.014^a^ (0.000)	−0.009^a^ (0.000)
Google Trend_t−1_	0.042^a^ (0.008)		0.044^c^ (0.052)		0.051^a^ (0.000)	0.064^a^ (0.005)	
Overlapping Google Trend_t−1_		0.035^a^ (0.004)		0.047^a^ (0.001)			0.057^a^ (0.000)
Market return_t−1_	0.008 (0.386)	0.004 (0.686)	0.013 (0.224)	0.005 (0.620)	0.002 (0.765)	0.001 (0.981)	−0.005 (0.497)
Volatility_t−1_	0.022^b^ (0.049)	0.024^b^ (0.023)	0.045^a^ (0.003)	0.032^a^ (0.006)			
Return squared volatility_t−1_					0.010^b^ (0.012)	0.036^a^ (0.000)	0.023^a^ (0.000)
Observation	4466	4466	4466	4466	4466	4466	4466
R-squared	0.228	0.247	0.201	0.237	0.212	0.183	0.227

The dependent variable is *Bubble*, which is equal to 1 during the bubble period and 0 otherwise. The lagged return is the 1-day lag of the cryptocurrency return. Volume is a 1-day lag in the trading volume. Google Trends is a 1-day lag of Google Trends search volume. Market return is the 1-day lag CCI index return and volatility is the 1-day lag of volatility. Amihud is the 1-day lag of the 30 days average of the price changes per dollar of the volume unit of trade. Overlapping Google Trends is the one-day lag of the Google Trends search volume, calculated using 115 overlapping data periods. Return-squared volatility is the 1-day lag of the 30-days rolling window standard deviation of the daily return. All variables were standardized. We used random effects and robust standard errors clustered by cryptocurrency in the panel estimation

R-squared refers to the McFadden pseudo R-squared value

a, b, and c represent the significance at the 1, 5, and 10 percent levels, respectively

## Conclusion

Bubble behavior in different investment instruments is frequently discussed in finance as bubbles are potential sources of financial instability. However, cryptocurrency bubbles, especially altcoins, require further investigation. Considering that total market capitalization of cryptocurrencies exceeds $2 trillion, our study attempts to detect cryptocurrency market bubbles and determine the factors behind them.

To achieve the aim of this study, we employed the PSY methodology to analyze bubble periods in Bitcoin, Ethereum, Ripple, Cardano, Dogecoin, Chainlink, VeChain, Litecoin, Stellar, Theta, and Tron during the COVID-19 period (January 1, 2020, and March 31, 2021). We then examined herding behavior in bubble periods by adopting the CSAD approach and analyzing co-explosivity among cryptocurrencies. In the last step, we estimated the determinants behind cryptocurrency bubbles by employing panel and time-series probit estimations using two sets of variables: (1) cryptocurrency-specific factors (lagged return, volume, and volatility) and (2) market-related factors (market return and Google Trends).

Both investors and policymakers focus on pricing cryptocurrencies. Investors are interested in cryptocurrencies because of their high return potential, whereas policymakers monitor the cryptocurrency market because a potential problem in this market could spread to the entire economy. Our study findings may provide a certain amount of information to both parties. Moreover, our results show that, although the number of bubble days differs among cryptocurrencies, all digital currencies present evident bubble characteristics during the COVID-19 period. Furthermore, co-explosivity analysis reveals that explosive price movement in one cryptocurrency leads to explosivity in those of others. Extant results show that investors can follow co-explosive price movements by switching one cryptocurrency to another to gain profit. However, an investor should also consider that, in time, the bubble might explode, and cryptocurrency price may plummet. Therefore, they should invest in different instruments for cryptocurrencies instead of diversifying portfolios among cryptocurrencies. The overall results suggest the need to regulate the cryptocurrency market. Given the increasing market share of cryptocurrencies in the financial market and poor financial literacy among cryptocurrency investors, cryptocurrency market bubbles threaten financial stability. Possible cryptocurrency bubble bursts along with the damage caused by the pandemic could seriously delay economic recovery. Therefore, although policymakers cannot regulate the cryptocurrency market because of its decentralized nature, they can develop appropriate practices (e.g., programs to increase cryptocurrency financial literacy) and information policies about cryptocurrency market bubbles and their possible outcomes.

Regarding herd behavior, we find that herding is common during the pandemic periods; however, adverse herding dominates during bubble periods, suggesting that herding is not related to the bubble. Given these findings, investors should be aware of herd behavior during normal periods and follow their beliefs in bubble periods. Policymakers should also increase information campaigns during normal periods to maintain herding awareness in the cryptocurrency market. Based on the results, both bubbles and herd behavior in cryptocurrencies highlight the inefficiency and necessity of regulating cryptocurrency markets. On factors behind speculative bubbles, volume and volatility among cryptocurrency-specific factors are positively associated with bubbles, and Google Trends is the only market-related factor positively associated with bubbles. Investors can use these variables to estimate bubbles in a particular digital currency and benefit from explosive price movements.

Future studies should consider the impact of the announcement of stimulus packages by governments during the pandemic on bubble and herd behavior in the cryptocurrency market. Additionally, pandemic periods may be divided into phases, and bubble and herd behavior in different phases should be examined. The post-pandemic period should also be investigated, and results should be compared with the findings of the present study.

## Data Availability

The datasets generated and analysed during the current study are available Cryptocurrencies Index 30 at (https://cci30.com), Bitcoin, Ethereum, Ripple, Cardano, Dogecoin, Chainlink, VeChain, Litecoin, Stellar, Theta and Tron Price Index are available at (https://coinmarketcap.com). Google trend data is downloaded from (https://trends.google.com/trends/?).

## Appendix

See Table

**Table 11:** Co-explosive analysis with control variables

	BTC	ETH	XRP	ADA	DOGE	LTC	LINK	VET	THETA	TRX
BTC_t−1_		0.087^a^ (0.002)	−0.067^b^ (0.019)	0.014 (0.700)	0.023 (0.393)	0.102^a^ (0.002)		−0.019 (0.519)	0.158^a^ (0.000)	−0.012 (0.670)
ETH_t−1_	0.213^a^ (0.000)		0.055 (0.207)	0.043 (0.325)	0.034 (0.135)	−0.029 (0.355)		−0.043 (0.284)	−0.203^a^ (0.000)	0.032 (0.315)
XRP_t−1_	−0.052 (0.534)	0.174^a^ (0.000)		−0.053 (0.596)	0.157^a^ (0.000)	0.059 (0.120)				
ADA_t−1_	−0.031 (0.443)	0.014 (0.574)	−0.037 (0.353)		−0.040 (0.118)	−0.059^b^ (0.025)		0.034 (0.411)	−0.067^c^ (0.083)	0.070^a^ (0.003)
DOGE_t−1_	0.053 (0.315)	0.047^c^ (0.078)	−0.040 (0.354)	−0.053 (0.253)		0.121^a^ (0.002)		−0.032 (0.488)	0.004 (0.951)	0.034 (0.147)
LTC_t−1_	0.235 (0.315)	0.082^b^ (0.015)	0.140^a^ (0.000)	0.098 (0.167)	0.099^a^ (0.000)			0.146^b^ (0.021)	0.202^b^ (0.010)	0.011 (0.784)
LINK_t−1_								−0.029 (0.575)	0.231^a^ (0.000)	0.087^a^ (0.002)
VET_t−1_	−0.068 (0.111)	−0.005 (0.910)	0.053 (0.113)	0.048 (0.227)	−0.101^b^ (0.012)	0.043^c^ (0.075)	0.048 (0.296)		0.013 (0.811)	−0.016 (0.688)
XLM_t−1_	−0.212 (0.247)	−0.273^a^ (0.000)	−0.053 (0.180)	−0.074 (0.463)	−0.177^a^ (0.002)	−0.078 (0.121)				
THETA_t−1_	0.106^a^ (0.000)	−0.077 (0.567)		−0.060^b^ (0.031)	0.024 (0.256)	−0.017 (0.420)	0.015 (0.584)	−0.012 (0.614)		0.016 (0.510)
TRX_t-1_	0.116^b^ (0.019)	−0.023 (0.567)	0.041 (0.276)	0.261^a^ (0.000)	0.063^b^ (0.025)	−0.041 (0.211)	0.036 (0.491)	0.006 (0.919)	0.191^a^ (0.001)	
Lagged return_t−1_	0.100 (0.151)	0.020 (0.344)	0.004 (0.518)	−0.007 (0.680)	0.002 (0.747)	0.013 (0.599)	0.058^b^ (0.033)	0.009 (0.510)	0.050^a^ (0.005)	0.003 (0.830)
Volume_t−1_	−0.114 (0.226)	0.343^a^ (0.001)	0.135^a^ (0.002)	0.218^a^ (0.000)	0.081^a^ (0.000)	−0.173^b^ (0.011)	0.156^a^ (0.000)	0.041 (0.431)	0.281^a^ (0.000)	0.003 (0.939)
Google Trend_t−1_	0.173^a^ (0.000)	0.018 (0.243)	0.001 (0.932)	0.016 (0.267)	−0.012 (0.538)	0.052^a^ (0.003)	0.037^a^ (0.000)	0.086^a^ (0.000)	0.068^b^ (0.026)	0.053^a^ (0.001)
Market return_t−1_	−0.045 (0.194)	0.013 (0.346)	−0.002 (0.641)	0.010 (0.562)	0.014^c^ (0.087)	−0.007 (0.633)	−0.035 (0.133)	0.001 (0.969)	0.000 (0.997)	0.003 (0.849)
Volatility_t−1_	−0.029 (0.387)	−0.045^a^ (0.001)	−0.006 (0.430)	0.027 (0.175)	0.012 (0.145)	0.028^b^ (0.038)	−0.019 (0.423)	0.003 (0.810)	0.008 (0.632)	0.023^a^ (0.008)
R-squared	0.401	0.609	0.504	0.506	0.668	0.537	0.256	0.286	0.423	0.433

a, b, and c represent the significance level of 1, 5, and 10 percent level, respectively

P-values are in the parenthesis

11.

## Abbreviations

S&P 500: Standard and Poor’s 500 Price Index
CCI: Cryptocurrencies Price Index 30
ETH: Ethereum price
XRP: Ripple price
ADA: Cardano price
DOGE: Dogecoin price
LINK: Chainlink price
VET: VeChain price
LTC: Litecoin price
XLM: Stellar price
THETA: Theta price
TRX: Tron price
BTC: Bitcoin price
BTCD: Bitcoin dominance index
PSY: Philips, Shi, and Yu model
SADF: Supremum augmented Dickey-Fuller model
BSADF: Backward supremum augmented Dickey-Fuller model
CSSD: Cross-sectional market deviation
CSAD: Cross-sectional absolute deviation
GARCH: Generalized autoregressive conditional heteroskedasticity
GSADF: Generalized supremum augmented Dickey-Fuller model

## Footnotes

1 For instance, Ethereum, Dogecoin, Cardano, Litecoin, and Theta reached their highest levels in the first quarter of 2021. Others, such as Ripple, Stellar, and Tron, were very close to testing the 2017 peak levels.

2 Chang (2014) proposes a mathematical explanation of the interaction between herd behavior, speculative bubbles, and social interactions. The fundamental equations in his study are formulated as follows:

1 $$x^{*} = \frac{{m^{*} b}}{{R - km^{*} }}$$

2 $$m^{*} = \tanh \left( {\beta J_{m} m^{*} } \right)$$

If the $\beta J < 1$, there is only one steady-state (0,0), however, if $\beta J \ge 1$, there can be more than one steady-state in asset prices. Arbitrageurs can make a profit if the steady-state asset price is higher than its fundamental value and make no profit if the steady-state price is equal to the fundamental price. Herding behavior exists in the steady-state ($m^{*}$, and $x^{*}$) if the binary belief choice $\left( {\omega_{i} } \right)$ has a similar sign as $m^{*} { }$ and $m^{*} \ne 0$. If all arbitrageurs are rational, the steady-state level is the initial stage and all investors’ anticipation of the average selection level at time *t* is $m_{t}^{e} = m_{t - 1}$. Social interaction causes herding behavior. Finally, the speculative bubble is the deviation from fundamental prices with a solution $x^{*} = 0$. If a strong social interaction ($\beta J > 1$) is present, the speculative bubble occurs in the initial steady-state which causes herding among investors. The price bubble remains in the new steady-state of the economy. The speculative bubble disappears in a short period when social interaction weakens. However, if the social interaction stays strong and the belief of investors is biased in the economy, price extrapolation continues and causes the speculative bubble to remain at the new steady-state, which suggests that investors continue engaging in herding behavior.

3 The Crypto Currencies Index is downloaded from the website: https://cci30.com/.

4 See Philips, Shi and Yu (2015) for detail information regarding to detecting bubbles procedure.

5 Co-explosivity analysis with the control variables provides similar results as in Table 8. For the results, see Appendix.

6 Correlation between the original 270 days data and estimated data is higher than 95% for each cryptocurrency.

## Code availability

STATA code is available upon reasonable requests.
